# Endometriosis in the Cecum: A Rare Clinical Entity

**DOI:** 10.7759/cureus.35782

**Published:** 2023-03-05

**Authors:** Ioanna Verzoviti, Dimitrios Kalliouris, Anastasia Boptsi, Nikolaos Kiriakos, Dimitrios Keramidaris

**Affiliations:** 1 Department of General Surgery, 417 Army Shared Fund Hospital, Athens, GRC; 2 Department of Gastroenterology, 401 Army General Hospital, Athens, GRC

**Keywords:** cecum, endometriosis, cecal endometriosis, bowel endometriosis, tumor-like lesions

## Abstract

Cecal endometriosis is uncommon and may mimic other tumors of the colon, making it difficult to safely diagnose preoperatively. We report a case of a 50- year-old female who was found to have a cecal lesion during an endoscopic examination, which was performed for the investigation of anemia. It was also confirmed by conducting a computed tomography (CT) scan. Due to the high possibility of this mass identification as a neoplasm, the patient underwent a laparoscopic right hemicolectomy with an extracorporeal side-to-side isoperistaltic anastomosis. However, the postoperative histological diagnosis of the mass was cecal endometriosis, as the histopathology report noted endometrial tissues in the submucosa and muscolaris propria of the ileocecal region. Endometriosis of the cecum is a rare manifestation and can often be misdiagnosed as a malignant tumor. Further research is required, concerning preoperative characteristics of bowel masses in women, in order to provide optimal operative treatment and avoid unnecessary invasive procedures.

## Introduction

Endometriosis, the presence of an ectopic functioning endometrial tissue outside the uterus, represents a benign condition and affects 6-10% of women in their reproductive age [1,2].

It can affect almost any organ or structure; however, the pelvic cavity is the most common location for endometriotic implants. Atypical endometriosis is rare and difficult to diagnose [3].

Gastrointestinal endometriosis is the most common form of extragenital endometriosis (EE). In nearly 90% of cases, it affects the rectum and sigmoid colon [4]. The small intestine follows, most commonly the ileum, in 7-12% of cases, and the appendix in 6-8% of cases [1]. The occurrence of cecal involvement is relatively rare and constitutes less than 3.6-6% of gastrointestinal endometriosis cases [1,2,5,6].

The second most common form of EE is urogenital endometriosis. It affects the bladder in more than 85% of cases. The diaphragm is the most common site of thoracic endometriosis. In abdominal wall endometriosis, painful nodules arise in scars from prior abdominal surgery [4].

Symptoms are nonspecific and vary according to the site of involvement, and they are usually abdominal or pelvic pain, nausea, vomiting, diarrhea, and rectal bleeding [7]. When it affects the appendix and/or ileum, it may cause clinical situations, such as acute appendicitis, perforation, or intussusception, and may be associated with menses [5,7]. Urogenital endometriosis might present with dysuria, hematuria, or irritable bladder syndrome. Endometriosis of the diaphragm may cause period-associated shoulder pain or catamenial pneumothorax. Endometriosis affecting a nerve often presents with sciatica [4]. Complete intestinal obstruction is not a common finding in patients with endometriosis [2].

The differential diagnosis of intestinal endometriosis is difficult, frequently resulting in delayed diagnosis and treatment. Often it can only be confirmed postoperatively [8]. Intestinal involvement of endometriosis often causes mass-like lesions and the malignancy can only be excluded postoperatively by histopathological examination [5,6]. Laparoscopy with biopsy remains the definitive method for diagnosis [9].

Roman et al. conducted a case series study concerning the surgical management of patients with deep infiltrating endometriosis of the rectum and the sigmoid colon. The cecum was involved in 6.6% of the cases [10].

We present a case of a postmenopausal female patient with cecal endometriosis. In our case, endometriosis was presented as a mass during diagnostic endoscopy.

## Case presentation

A 50-year-old female, gravida 2, para 2, with a family history of colon cancer, was referred to our hospital due to anemia (hematocrit: 29%). From her medical history, it was found that she had undergone a prior curettage, an exploratory laparoscopy for endometriosis, 25 years ago, which was negative, and an open appendicectomy 14 years ago. The patient was totally asymptomatic and the rest of the blood tests were within normal limits. 

**Figure 1:**
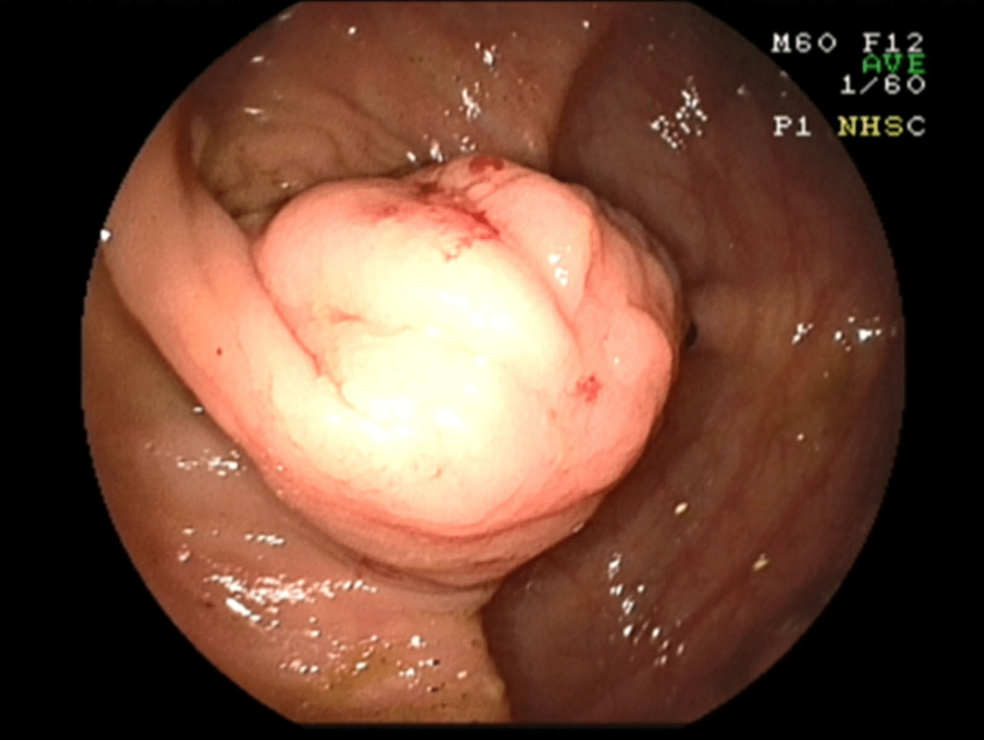
Colonoscopy demonstrating the cecal mass.

An endoscopic examination of the large bowel was requested from the gastroenterology department in order to investigate her anemia. It revealed a cecal hard mass of 2.5-3 cm, which seemed to be more of submucosal or parietal origin, while the bowel’s mucosa seemed to be intact with no evidence of lesion. Biopsies were taken and the histology report was indicative of normal colonic mucosa (Figure 1).

The patient was referred to our surgical department for further investigation. To clarify, a computed tomography (CT) scan of the abdomen was performed and confirmed the presence of a mass, 2.8 x 3.2 cm, without any lymph node pathology.

Based on all the aforementioned findings and because the patient had a family history of colon cancer, she underwent a laparoscopic right hemicolectomy with an extracorporeal side-to-side isoperistaltic anastomosis to remove the entire mass. Intraoperative inspection revealed no obvious implants or abnormalities of the pelvic or the upper abdomen.

**Figure 2:**
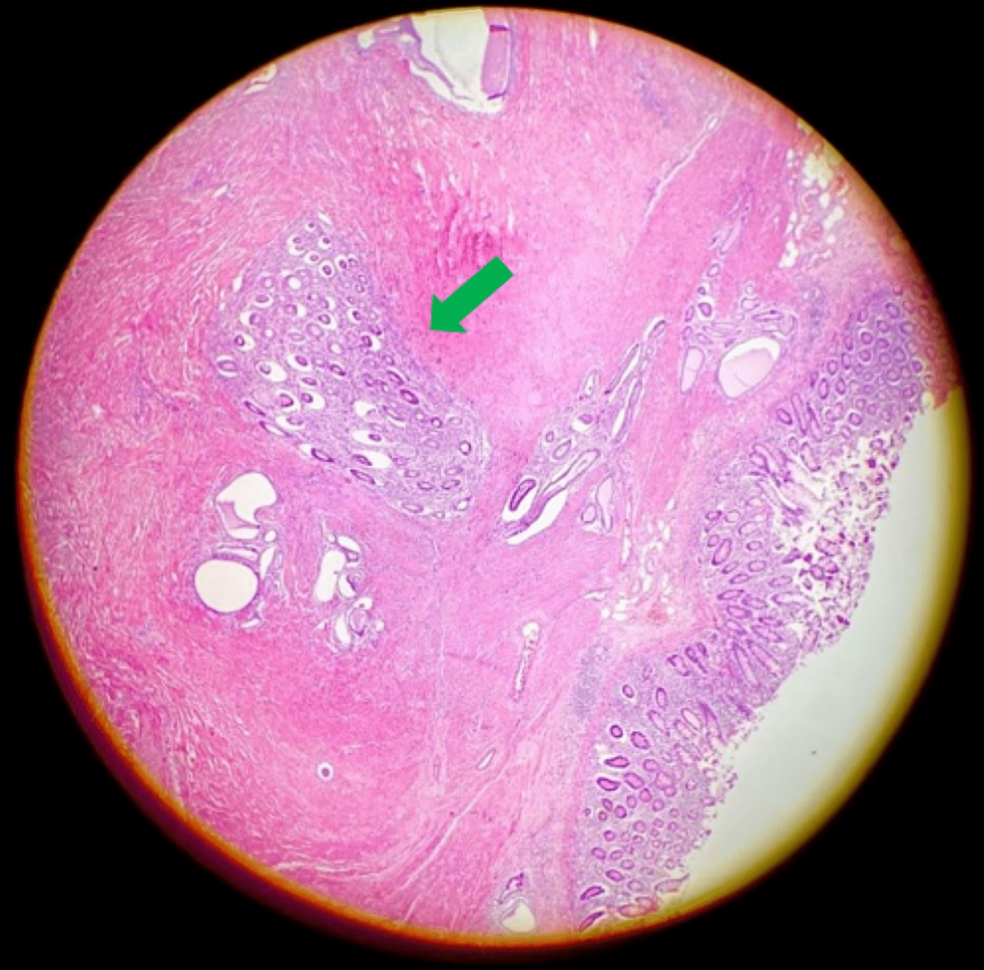
Endometrial tissue (green arrow) inside normal colonic submucosa.

**Figure 3:**
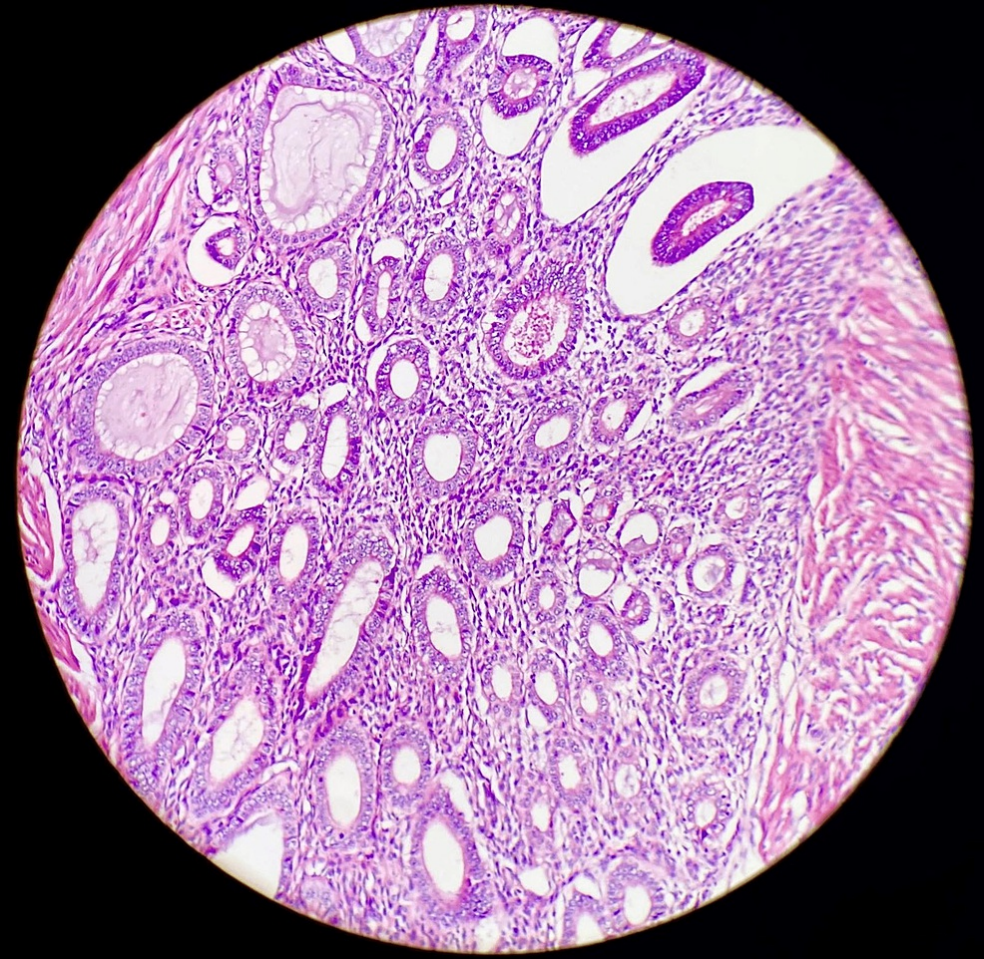
Magnification of the endometrial tissue.

**Figure 4:**
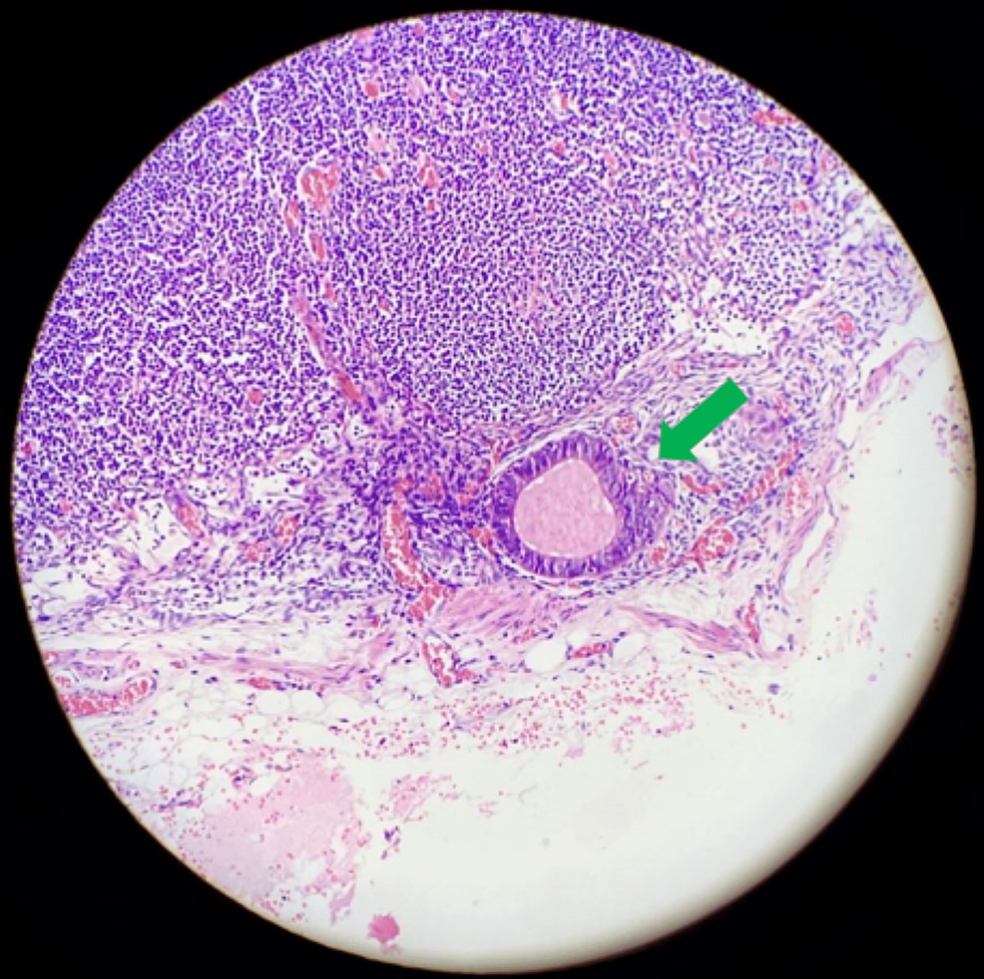
The green arrow indicates an endometrial gland inside normal lymphatic tissue.

The final histological examination of the specimen revealed endometrial tissues in the colonic submucosa and muscolaris propria of the ileo-cecal region, with negative cytology for malignant cells (Figures 2-4).

The postoperative period was uneventful and the patient was discharged from our clinic on the sixth postoperative day. The patient was followed up at the first, third, sixth, and ninth months with no findings in clinical and imaging examination.

## Discussion

Women with bowel endometriosis are often diagnosed with other disorders such as irritable bowel syndrome, inflammatory or ischemic colitis, diverticulitis, malignancy, and pelvic inflammatory disease [1]. In this case, however, the patient was totally asymptomatic.

To date, there are no clear guidelines on the evaluation of patients with suspected bowel endometriosis.

Different imaging modalities have been proposed including transvaginal and/or transrectal ultrasonography, magnetic resonance imaging, and double-contrast barium enema. These techniques provide useful information regarding the presence, location, and extent of endometriosis [11].

Transvaginal sonography techniques have a sensitivity and specificity of 79% and 94%, respectively. As far as magnetic resonance imaging (MRI) is concerned, 94% sensitivity and 77% specificity were observed. For rectosigmoid endometriosis, pooled sensitivity and specificity of MRI were 92% and 96%, respectively [12].

The gold standard diagnostic method is laparoscopic surgery with pathological confirmation [8].

Combined hormonal contraceptives with or without nonsteroidal anti-inflammatory drugs are first-line options for managing symptoms. Second-line treatments include gonadotropin-releasing hormone (GnRH) receptor agonists, GnRH receptor antagonists, and danazol. Aromatase inhibitors are reserved for severe diseases. All of these treatments seem to be effective but might cause several side effects [9].

As far as bowel endometriosis is concerned, there is no indicated treatment. Although endometriosis can be the origin of Mullerian tumors in women, a similar situation has not been described in intestinal endometriosis yet, due to the small number of cases of bowel endometriosis. However, it is theoretically possible for a cecal mucinous tumor to arise concurrently from intestinal endometriosis with metaplasia [5].

The Japan Society of Obstetrics and Gynecology and the Japan Society of Endometriosis published guidelines concerning the treatment of EE [13].

As far as rectosigmoid endometriosis is concerned, medical treatment has been reported to improve symptoms and reduce the lesion size but did not appear to be superior to surgical treatment. For intestinal endometriosis involving the cecum, there are no reports that assess the efficacy of medical therapy [13].

There are no guidelines specifying which lesions should be operated on and which standardized surgical technique is recommended. For this reason, the therapeutic option should be tailored according to the patient's symptoms. Especially in cases of bowel obstruction, surgical treatment is effective in improving pain [11].

Fu et al. suggested that in any ileocecal lesion with mucinous epithelium or mucin extrusion, the possibility of a cecal mucinous tumor arising from endometriosis with intestinal metaplasia must be considered [5].

Surgical treatment should also be chosen when endometriosis affects the appendix since the risk of secondary intussusception of other bowel segments is high [14].

Laparoscopy is the ideal option as it allows precision and complete assessment of the peritoneal cavity. It is also a well-tolerated and feasible technique [11,15].

Patients who are not candidates for surgical treatment should receive medical treatment. First-line therapy comprises the long-term use of oral contraceptives. There are also several other medications available to manage bowel endometriosis that actually aim to reduce circulating hormones. Generally, there is a rationale for medical treatment before surgery to improve the patient’s symptoms, potentially negating the need for surgical intervention [11].

In our patient, there was no suspicion of endometriosis. Malignancy was suspected due to the incidental mass-like lesion and her family history of colon cancer. However, the final diagnosis could only be given by the histopathological examination, which confirmed endometriosis involving the cecum without any evidence of malignancy.

## Conclusions

Endometriosis of the cecum is a rare clinical entity and presents diagnostic challenges, as limited information is available in the literature. It is essential to include in our differential diagnosis rare diseases that imitate symptoms of patients, who often come to the hospital with the suspicion of colon cancer. Preoperative examination of the characteristics of bowel tumors in women could be recommended, as cecal endometriosis might erroneously be diagnosed as a malignant tumor. To date, there are no clear guidelines on the diagnostic and therapeutic options of cecal endometriosis. Due to the limited reported cases, optimal management strategies are yet to be identified.
